# The Tutor of Resilience Program with Children Who Have Experienced Maltreatment: Mothers’ Involvement Matters

**DOI:** 10.1007/s10578-022-01393-w

**Published:** 2022-08-02

**Authors:** F. Giordano, C. Taurelli Salimbeni, P. Jefferies

**Affiliations:** 1 Department of Psychology – Resilience Research Unit, Università Cattolica del Sacro Cuore Largo Gemelli 1, Milan, MI 20121 Italy; 2 Family and Community Resilience, Canada Research Chair in Child, Resilience Research Centre Dalhousie University, PO Box 15000, Halifax, NS B3H4R2 Canada

**Keywords:** Resilience, Intervention, Child maltreatment, Trauma-related outcomes, Parental involvement

## Abstract

Resilience is a dynamic process involving the presence and interaction of personal and environmental factors that modify the impact of adversity. Resilience-building interventions are therefore important for improving trauma-related outcomes in children and caregivers exposed to adversity. This study examines the impact of the Tutor of Resilience (TOR) program on beneficiaries’ trauma-related symptoms and on mother–child interactions in a group of children exposed to maltreatment (*N* = 186; mean age = 11.95; SD = 2.50). Assessments were completed at baseline and post-intervention. RM-ANOVAs indicated significant improvements for most trauma symptoms (anxiety, anger, post-traumatic stress, and disassociation, but not depression) in the intervention group relative to a control group (*N* = 88; mean age = 10.76; SD = 2.57), and indicated further improvements to anxiety and dissociation for the intervention group when mothers were involved. Mother–child interactions also improved over time, as did their overall trauma symptoms and distress. Findings support the effectiveness of the ToR, especially when involving mothers.

## Introduction

The Regional Index of child maltreatment edited by the CESVI foundation is an important national report which provides data on children’s vulnerability in Italy. In 2020, it revealed that maltreatment continues to be a serious problem [1]. Nearly five percent of children (47.7 out of 1,000) are taken into care by social services. The Centers for Disease Control and Prevention (CDC) have defined maltreatment as “any act or series of acts of commission or omission by a parent or other caregiver that results in harm, potential for harm, or threat of harm to a child” [2], and indeed, approximately 100,000 Italian children (about 9.5 per 1,000 residents) were found to have been exposed to different types of adverse childhood experiences (ACEs), which reflect these acts of commission and omission [1]. The report also notes a clear difference between the North and South of Italy, which may be attributable to the lower levels of social services and the higher presence of risk factors in the South.

The impact of maltreatment on a child's mental health and wellbeing is well-known: chronic experiences of physical, psychological, and sexual abuse are associated with symptoms of depression [3], anxiety disorders [4], interpersonal difficulties [5], and an increased risk of suicide attempts in adulthood [6]. More generally, studies show that adverse childhood experiences are one of the primary predictors of trauma-related symptoms [7, 8]. However, not all children exposed to maltreatment go on to experience negative outcomes [9]. Studies have indicated that between 20 and 50% of children exposed to maltreatment manage to achieve resilient functioning [10]. Resilience is conceptualized as a dynamic process that involves drawing on both internal and external resources to achieve positive outcomes despite adversity [11, 12]. It also refers to the absence of mental health or psychosocial problems despite severe hardships [11]. A growing number of studies recognize resilience as an ecological phenomenon which is developed through interactions within the environment, including supportive families, schools, neighborhoods, and the larger community [13, 14]. Therefore, the development of research-informed resilience-building interventions have been increasingly considered critical for preventing trauma-related outcomes in individuals exposed to adversity [15, 16].

Several resilience-focused interventions have been developed for children who have experienced maltreatment [17], war [18, 19], and other kinds of adversity [20, 21]. Several studies have demonstrated the efficacy of these resilience-enhancing programs in terms of symptom reduction in target beneficiaries [22, 23]. However, rather than focusing on psychological maladjustment, these interventions reinforce children’s strengths, in terms of their inner resources such as coping strategies [24], emotional self-regulation [25], self-reinforcement [26], self-efficacy [27] and social resources, such as parental support [28, 29] and other supportive relations [30, 31].

With regard to the latter social resources, several studies affirm that resilience processes are facilitated by protective relationships [32] and physical ecologies that make resources available and accessible in ways that individuals experience as meaningful [33]. Indeed, whether formal or informal, supportive relationships have been shown to exert a remarkable effect on outcomes, especially for children and youth exposed to violence [34, 35]. Cultivating positive environmental contexts within families, schools, social services, and communities may therefore counteract risks in children’s lives [36].

In particular, family is the main caregiving environment thought to have the greatest impact on the development of resilience in children [13]. Indeed, strong family support networks have been associated with lower levels of aggressive behaviors in children exposed to maltreatment [37]. Based on the premise that child development and mental health is shaped by parenting [38], several studies have shown the benefit of including parents in children support programs in order to enhance assets and resources [39], prevent and reduce negative outcomes by decreasing parental distress [40]. Other studies show that the inclusion of parents in children support programs may lead to improvements in terms of reduced trauma-related symptoms and other mental health outcomes in both parents and children [41, 42] as well as increased positive child-parent interactions [43].

Furthermore, formal service providers who are trained to offer safe, stable, caring and encouraging professional relationships with program beneficiaries can enhance the likelihood of children’s positive outcomes over time [17, 44]. In particular, research involving children who have experienced maltreatment highlights that interventions by social welfare and education service providers delivered through government and non-governmental organizations may shape opportunities to recover [45]. However, social providers cannot serve as effective resilience-building actors if they are inadequately trained in approaches to resilience-building [46, 47]. To that end, the *** of the *** designed the Tutor of Resilience model for psychosocial care [48, 49], which aims to guide service providers in the creation of culturally and contextually sensitive interventions in ways that both mitigate risk and enable access to resilience-promoting resources for children, youth or families experiencing one or more forms of adversity, thereby enhancing their psychosocial well-being.

## The Tutor of Resilience Program

The Tutor of Resilience (ToR) is a transnational model of psychosocial care developed to increase the skills of professional helpers to act as resilience-enablers (*see* 48, 49 for worked examples). It is built on the premise that resilience is a social-ecological process that helps individuals navigate and negotiate for personal and collective resources through interpersonal relationships that increase access to psychosocial and physical supports [33, 50]. The ToR is rooted in large multisite action-research projects and interventions conducted by the Resilience Research Unit of the Catholic University of Milan, in national and international contexts [15, 51–54].

**Fig. 1:**
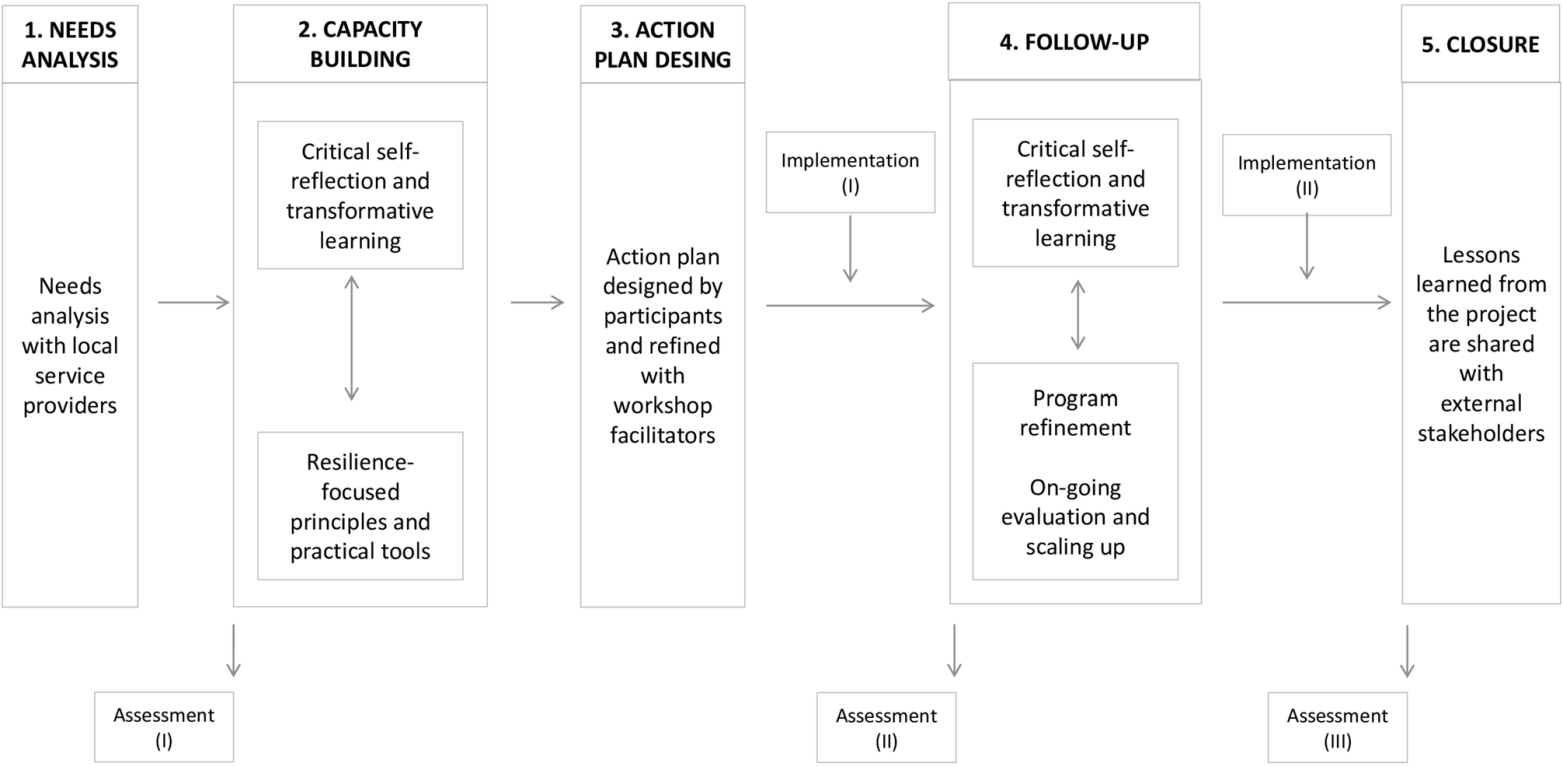
The five phases of the Tutor of Resilience model. Reprinted from Ref. 48. Copyright (2021) by Frontiers

When the ToR model is used to develop a program, it involves five phases (Fig. 1): a needs assessment, capacity building, the action plan design which guides the first implementation, a follow-up, followed by the second implementation, and the program closure.

In the first phase, *needs assessment* meetings have the goal of defining the psychosocial needs and barriers to service experienced by the target beneficiaries, as well as identifying the most relevant protective factors and processes that may help them to manage their difficulties. A process of personal reflection and small and whole group reporting ensues before consensus is sought regarding the most important challenges and potential sources of support.

The *capacity building* phase is divided into a set of modules which are designed to help train staff who will conduct the program.

- Module I: *the psychosocial approach to support and maintain resilience*, focused on the meaning of empowerment, resilience and beneficiaries’ personal and collective potential for recovery;
- Module II: *the psychological trauma in children exposed to adversity and its interaction with multilevel developmental processes*, which provides a comprehensive summary of psychological trauma and the underlying mechanisms through which trauma affects the identity formation of beneficiaries and their functioning;
- Module III: *Identification, prevention and appropriate responses to beneficiaries who have experienced adversity*, which shared new tools that providers could adapt for their work with children and families at risk, conceived as ingredients in a grocery store which can be assembled in any number of different combinations to produce a meal (in this case, a localized curriculum) (*see* 55];
- Module IV: *Self-confrontation and critical reflection on relationships that support resilience*, where trainees were asked to create meaning out of their past experience as a service provider and what it would mean to be a Tutor of Resilience with their target beneficiaries;
- Module V: *Monitoring the implementation of the ToR program*, where a monitoring plan was developed and refined with participants.

Each module involves sharing some of the relevant knowledge and evidence related to issues identified in the needs assessment, but no specific activities are suggested as interventions at this stage. A combination of didactic presentations, hands-on interactive exercises, and case studies are employed for trainees to discover different ways they can design and conduct interventions for their beneficiaries. At the end of the capacity building phase, participants are encouraged to reflect on the following evidence-informed resilience-enabling principles which come from practices that have been shown to enhance wellbeing among children who have gone through adversity:

- Widen the participants’ perspective of the beneficiaries, so that it is not limited to addressing problems and difficulties and instead focuses on the beneficiaries’ strengths [27].
- Help beneficiaries discover their own internal resources and talents and reinforce them. In particular, the following resources have been taken into consideration and are amenable to improvement in well-designed ToR programs: self-efficacy [55], self-awareness [56], projecting oneself into a meaningful future [57], coping abilities [58, 59], and social skills [15, 60].
- Enhance beneficiaries’ emotional competence [61] and emotional regulation [62] in order to mitigate negative consequences of stress [44] and decrease emotional reactivity [63], as these are thought to have an adverse effect on psychosocial development [17].
- Reinforce beneficiaries’ relationships with peers [64, 65], and service providers [45] to help develop trust in others.
- Strengthen family systems by enhancing family cohesion [58, 66] and communication [65], and creating stronger family support networks [64, 67] that improve caregiving [28].

Trainees are then challenged to design activities fitting with the needs identified in the first phase while also adhering to these principles.

A Tutor of Resilience *action plan* reflecting the resilience-enabling principles is then designed by trainees to be implemented with their target beneficiaries and submitted for review to the workshop facilitators who work individually with each trainee to refine the plan if necessary. A two-day *follow-up* workshop is then arranged for six months after the first workshop. This follow-up workshop is based on the four levels of training evaluation criteria included in the Kirkpatrick model of training evaluation [68]: reactions, learning, behaviour, and results. The workshop includes an ongoing evaluation and leads to program refinement and the revision of an action plan, incorporating lessons learned from the first iteration, that guides the second implementation.

The *closure* stage consists of a two-day meeting with project staff, aimed to identify lessons learned from the project as a whole. Trainees are invited to reflect on what they consider fundamental methods and messages that help to form an effective resilience-focused intervention in settings like theirs. These reflections from the field are shared with external stakeholders working with similar target beneficiaries. Recommendations for further refinement of the model and how best to assess outcomes are also discussed during this final meeting, with input drawn from the periodic assessments carried out with trainees.

While the ToR model has been described in previous studies [48, 49] to our knowledge no study has been conducted to test the impact of ToR on the mental health and wellbeing of children who have experienced maltreatment. Furthermore, the inclusion of non-abusive caregivers in children’s support programs is known to lead to improvements in the wellbeing of both children and parents wellbeing as well as the quality of their interactions, but to our knowledge, no study has examined the parental involvement in resilience-building interventions with children who have experienced maltreatment.

However, the ToR program didn’t involve all the children’s caregivers, as their participation in the ToR program was voluntary. Caregivers who decided not to get involved could still access to the center as normal, where they could ask any questions or voice concerns about their children or the program.

The aim of the study was, therefore, to investigate the impact of the ToR program^1^ with a group of children who have experienced intrafamilial or extrafamilial maltreatment, with and without the involvement of non-abusive caregiver. Since the present study only involved mothers of children who had experienced maltreatment (*see* later), we have worded the following hypotheses accordingly. In particular, we hypothesized that:

- There would be a significant decrease in trauma-related symptoms in children taking part in the ToR program, when compared to a control group.
- Parents involved in the ToR program activities would also report significantly lower levels of trauma-related symptoms; furthermore, they would report significantly increased positive interactions with their children and a reduction in distress.
- A differential treatment effect would be found with respect to the involvement of mothers in the ToR program. In particular, children whose mothers were involved in the ToR activities would report a decrease in trauma-related symptoms, compared to children who took part in the program but whose parents were not involved.

## Method

### Participants

The study was conducted in three Italian day-care centers in Bari, Bergamo, and Napoli. The sample consisted of 274 children between the ages of 7 and 18 (*M* = 11.95, *SD* = 2.50). The intervention group consisted of 186 children (102 girls, 84 boys). Among them, 40.5% had been exposed to neglect, 37.3% to parental conflict, 20% to domestic violence, 8.1% to physical violence, 3.2% to educational neglect and 2.2% to sexual abuse. These proportions are similar to reported national prevalence rates [65] (*see* Table 1). The intervention group was evenly distributed among the day-care centers (Bari = 40%, Bergamo = 27%, Napoli = 33%). Participants were either self-referred to the day-care centers or referred from child protective services, child advocacy centers, and schools, due to their personal histories of intrafamilial or extrafamilial abuse.

**Table 1:** Characteristics of the study participants

Variables	Categories	Intervention (*n* = 186) N (%) or M (S.D.)	Control (*n* = 88) N (%) or M (S.D.)	National estimate (CISMAI, 2015)
Gender	Female	102 (45.4%)	35 (40.7%)	–
	Male	84 (54.6%)	51 (59.3%)	
Age (years)	–	11.95 (2.50)	10.76 (2.57)	–
Traumatic experiences	Neglect	75 (40.5%)	42 (47.7%)	47.1%
	Parental conflict	69 (37.3%)	19 (22.9%)	–
	Domestic Violence	37 (20.0%)	24 (28.9%)	19.4%
	Physical Violence	15 (8.1%)	10 (12%)	6.9%
	Educational abuse	6 (3.2%)	2 (2.3%)	–
	Sexual abuse	4 (2.2%)	3 (3.6%)	4.2%

Although 119 parents reported being involved in ToR activities proposed by the day care centers, only 69 parents of children in the intervention group provided data. Furthermore, in line with most studies that discuss parental involvement [69], those involved in the ToR were exclusively mothers. Among them, 9.2% had psychological issues and 14.5% had been exposed to traumatic experience in their life. 8.4% reported alcohol addiction and 6.7% reported a drug addiction. Finally, 13.4% of these mothers currently face justice problems, 17.8% had been on house arrest (a measure by which a person is confined by the authorities to their residence) and 11.8% had been in jail.

The control group consisted of 88 children, 40.7% of whom were girls and 59.3% boys. They came from a day-care center in Bari and would be invited to participate in the ToR program the following year, after the pilot study had concluded (a waitlist control design). They were aged 6–17 (*M* = 10.76, *SD* = 2.57) and had been exposed to one or more ACE. Both groups were similar in terms of their age range, the proportion of males and females, and the number of traumatic experiences (Table 1).

### Procedure

The study involved social workers, educators, psychologists, children, and parents of three day care centers who specialized in assisting children exposed to maltreatment operated by the “Consortium Fa” in Bergamo, the cooperative “Il Grillo Parlante” in Naples, and the “Giovanni Paolo II Onlus” foundation in Bari. The program was conducted in collaboration with CESVI Foundation, an Italian humanitarian organization that support vulnerable populations in promoting human rights. It lasted 13 months, from September 2018 until October 2019. Trainees attended 25 h of initial training over four days, and at the end they were provided with four intervention principles to pursue (see below). Six months after the initial training, in April 2019, trainees completed a two-day follow-up to monitor and refine the program, building on lessons learned during the first period of program implementation.

In the needs analysis, the lack of a shared framework and standards among the staff for how to build a supportive relationship with beneficiaries, as well as a need for tools and interventions tailored to families (specifically mothers) of beneficiaries, were highlighted by service providers as the most important needs. A ToR capacity building workshop was then developed and delivered to 50 educators and social workers working in the three day-care centers. At the end of the initial training, participants designed a ToR Action Plan based on the resilience-enabling principles discussed during the workshop (*see* in “Introduction”) to be implemented with their target beneficiaries. The action plan consisted of activities/actions through which each participant intended to follow each of the resilience-enabling principles. The action plans designed by participants are supervised by RiRes trainers, in order to check whether they understand the meaning of each principle, and that they have identified appropriate methods to pursue them. The action plan guided the program implementation. Part of the action plan included resilience workshops addressed to children in the day-care centers. When meeting children, participants were invited to select the resilience-enabling principles most relevant to them, and propose activities to follow the selected principles. Parents were invited to participate in support groups and in joint parent–child activities designed and delivered by service providers. Their participation was not mandatory. Two types of activities were conducted with mothers: self-care sessions for groups of mothers, aimed at addressing the sources of negative or unhelpful attitudes and helping them develop positive parenting skills and greater emotional literacy (improving their own emotional competence, enhancing their self-care, strengthening their confidence and building parental connectedness among participants). In parallel, mother and child workshops were run to guide mothers in assuming the role of tutor of resilience for their child through: strengthening their relationship with the child and improving their communication with their children to better understand their needs and foster more respectful and positive interactions, which are intended to support children’s development and build their resilience.

A two-day follow-up meeting was held with ToR trainers five months after the training, to reflect on the strengths, weaknesses, advantages and limitations of the ToR implementation and to revise a new Action Plan for the second phase of the implementation. A control group was recruited from a day care center located in Bari, which also hosted children who had experienced various forms of maltreatment.

Parents signed a consent form allowing their children to participate in the program and the accompanying evaluation study. Data were collected at three different moments: the first at baseline in October 2018, just prior to their participation in the program (pre-test), the second in February–March 2019 (for internal progress monitoring), and the third at the end of the program in July–September 2019 to track the impact of the model on beneficiaries’ mental health and wellbeing (post-test). Data regarding the pre-test and the post-test are taken in consideration in the current paper. Questionnaires were administered to the control group only at the pre- and post-test moments. Children completed the questionnaires with the assistance of the day-care center psychologists who were on standby to clarify any uncertainties when responding. The independence between teams responsible for research evaluation (i.e., psychologists) and program implementation (i.e., educators and social workers) minimized potential desirability bias in participant responses.

Participation in the study was voluntary, and no financial, monetary, or other incentives were provided. Participants were given the right to withdraw from the study at any time and without giving any reason. Mothers involved in the activities of the program were asked to complete questionnaires before and after the program. They took their questionnaires home to do this, but only 54% returned both pre- and post-administration questionnaires.

### Measures

The Trauma Symptom Checklist for Children (TSCC-A) [70] was used to measure child trauma-related symptoms. It is a questionnaire aimed at assessing the effects of childhood trauma through a child's self-report of trauma-related symptoms. The TSCC-A comprises 44 items which yield a score for six clinical subscales (anger, anxiety, depression, dissociation, and posttraumatic stress) and a trauma total score of the five factors. Items reflect symptom occurrence and are responded to on a 4-point scale ranging from 0 (“never”) to 3 (“almost all the time”). Scores were derived from the sum of subscale item responses, where higher scores reflect greater symptomatology (e.g., higher levels of anxiety). The instrument has been validated and translated into Italian [71]. In the present study, the subscales were found to have good internal consistency (anger: α = 0.83; anxiety: α = 0.79; depression: α = 0.81; dissociation: α = 0.81; posttraumatic stress: α = 0.81).

To measure adults’ trauma-related symptoms, the Parenting Stress Index and the Trauma Screening Questionnaire were administered. The short form of the Parenting Stress Index (PSI-SF) [72] is a 36-item questionnaire measuring stress levels within the parent–child relationship. Items are responded to on a five-point Likert scale, from 1 (“strongly agree”*)* to 5 (“strongly disagree”). The questionnaire yields a Total Stress score from three subscales: Parental distress, Parent–child dysfunctional interaction, and Difficult child, which assesses the parent’s view of the child’s temperament, defiance, noncompliance, and demandingness. The questionnaire has been validated and translated into Italian [73]. All subscales demonstrated good reliability in this study (parental distress: α = 0.83; parent–child dysfunctional interaction: α = 0.80; difficult child: α = 0.84). The Trauma Screening Questionnaire [74] is a 10-item adult screening tool. Parents were asked whether or not they had experienced each symptom at least twice in the past week. The TSQ items are answered by ticking ‘yes’ (symptom is present two times a week or more) or ‘no’ (symptom is not present or present less than twice a week); The total score indicated the level of PTSD reactions at the time the respondent completed the survey. The minimum score is zero and the maximum score is 10. The scale demonstrated good reliability (α = 0.84). The instrument did not exist in an Italian version; therefore, it was independently translated in Italian by a professional translator. The integrity of the items was then verified using the back-translation technique [75]. Discrepancies with the original English version were noted, and the Italian version was adjusted accordingly.

### Analysis

We compared children’s scores on the various outcome measures at T1 and T3 (T2 data was for internal progress monitoring) using a repeated-measures ANOVA to contrast potential improvements over time in trauma symptomatology between the intervention and control groups (a group × time interaction). As there was no control group equivalent for parents, a paired samples t-test was used to determine improvements in the trauma scores and parenting stress scores of the parents. Finally, to determine whether improvements in the intervention were due to parental involvement, a repeated-measures ANOVA was used to compare trauma scores of children whose parents were involved in the intervention with those who were not. All analyses were conducted in SPSS v25 [76].

## Results

The repeated measures ANOVA for trauma symptomatology indicated a significant improvement for all forms of trauma symptoms in the intervention group relative to the control group, except for depression (Table 2). Out of these improvements, anger presented the largest change (*F*(1,170) = 9.99, *p* = 0.020, *η*^2^_*p*_ = 0.056; moderate effect size), where intervention group participants reported a greater reduction in anger symptoms (Δ*M* = -2.11) compared to the control group (Δ*M* = 1.05). The smallest (yet still significant change) was found in dissociation scores (*F*(1,170) = 6.59, *p* = 0.028, *η*^2^_*p*_ = 0.037; small effect size), where the intervention participants again reported a greater reduction in symptoms (Δ*M* = -1.26) compared to the control group (Δ*M* = 1.39).

**Table 2:** Descriptive statistics and group tests of child-reported trauma symptom scores for intervention (*n* = 130) and control (*n* = 42) groups

	Pre-test		Post-test		Group comparison		
	Intervention *n* = 168 M (SD)	Control *n* = 83 *M* (SD)	Intervention *n* = 131 M (SD)	Control *n* = 43 *M* (SD)	*F*	*p*	*η*^2^_p_
Anxiety	8.67 (5.19)	8.07 (5.51)	6.71 (4.40)	8.57 (4.86)	7.16*	.028	.040
Depression	8.53 (5.13)	8.50 (5.27)	7.12 (4.93)	7.79 (4.78)	.51	.500	.003
Anger	9.33 (6.21)	7.05 (4.87)	7.22 (5.53)	8.10 (5.79)	9.99*	.020	.056
Post-traumatic stress	11.06 (5.45)	11.02 (5.43)	9.71 (5.39)	12.38 (4.96)	6.62*	.028	.037
Dissociation	9.22 (5.17)	8.90 (5.00)	7.96 (5.56)	10.29 (4.20)	6.59*	.028	.037

*p < .05; *F* = Repeated measures ANOVA test result; *η*^2^_*p*_ = partial eta squared. All p-values adjusted using the Benjamini–Hochberg method to cater for multiple tests/a greater incidence of false positives

The paired-samples t-test revealed that trauma scores improved for parents in the intervention group over time (*t*(69) = 3.44, *p* = 0.002) (Δ*M* = -1.16). Parenting stress scores similarly improved across all three subscales: parental distress (*t*(68) = 3.96, *p* < 0.001), parent–child dysfunction interaction (*t*(67) = 4.76, *p* < 0.001), and difficult child (*t*(67) = 2.35, *p* = 0.020) (Table 3).

**Table 3:** Descriptive statistics and group tests of parent-reported measures for the intervention group, using paired samples comparisons

	Pre-test	Post-test	Comparison	
	*n* = 155 M (SD)	*n* = 72 M (SD)	*t*	*p*
Trauma total score (*n* = 69)	4.23 (3.05)	3.10 (2.98)	3.33*	.002
Parental distress (*n* = 69)	31.59 (10.97)	26.50 (8.04)	3.89**	< .001
Parent–child dysfunction interaction (*n* = 68)	31.04 (11.62)	24.53 (7.32)	4.67**	< .001
Difficult child (*n* = 68)	32.49 (8.73)	29.53 (8.61)	2.39*	.020

*p < .05; **p < .001; *t* = paired samples t-test result. All p-values adjusted using the Benjamini–Hochberg method to cater for multiple tests/a greater incidence of false positives

Finally, when exploring the impact of parental involvement, anxiety, depression, and dissociation scores were found to improve over time for the children whose parents were involved in the intervention, compared to those who were not (*p*s < 0.05, *η*^2^_p_s = 0.036-0.042) (Table 4). However, only symptoms that significantly differed in the first test between intervention and control group were taken in consideration, therefore we excluded depression. No significant differences were found for anger and post-traumatic stress (Table 4).

**Table 4:** Descriptive statistics and group tests of child-reported trauma symptom scores for parents involved in the intervention (*n* = 78) and parents not involved in the intervention (*n* = 52)

	Pre-test		Post-test		Group comparison		
	Parent involved M (SD)	Parent not involved M (SD)	Parent involved M (SD)	Parent not involved M (SD)	*F*	*p*	*η*^2^_p_
Anxiety	9.10 (5.42)	8.02 (4.79)	6.32 (4.19)	7.29 (4.67)	5.39	.037	.040
Depression	8.87 (5.06)	8.02 (5.23)	6.55 (4.20)	7.98 (5.79)	5.67	.037	.042
Anger	9.51 (6.11)	9.06 (6.41)	7.12 (5.47)	7.38 (5.66)	.46	.500	.004
Post-traumatic stress	11.71 (5.39)	10.10 (5.50)	9.63 (5.22)	9.83 (5.67)	2.75	.125	.021
Dissociation	9.68 (4.97)	8.52 (5.44)	7.50 (4.93)	8.65 (6.38)	4.83	.043	.036

*p < .05; *F* = Repeated measures ANOVA test result; *η*^2^_*p*_ = partial eta squared. All p-values adjusted using the Benjamini–Hochberg method to cater for multiple tests/a greater incidence of false positives

## Discussion

This study illustrates the outcomes of a resilience-building program initiated to alleviate trauma-related outcomes in children who have experienced maltreatment. The program modeled several elements thought to be key features of successful psychosocial interventions. Specifically, it offered a group-based program with an emphasis on flexibly building contextually specific interventions; it was delivered by well-trained and supervised providers, and it involved family engagement.

Our findings indicate that the intervention led to improved mental health for participants. In particular, the results show that children involved in the ToR program reported a significant reduction in most of the trauma-related symptoms (anxiety, anger, post-traumatic stress, and dissociation) compared to the control group. The effect sizes helped to interpret these results, suggesting that the intervention generally had a small to medium effect on individuals. The decrease in most trauma-related outcomes in the ToR group suggests that strengthening emotional competence as well as processing the emotional content of trauma and developing a sense of purpose in life is beneficial to individuals [77]. Previous studies of resilience-focused interventions conducted with children who have experienced maltreatment [17] and other types of adversity have similarly reported significant decreases in anxiety [62], dissociation [78] anger and post-traumatic stress [79, 80].

Our study did not find a significant difference in the reduction of depression symptoms in the ToR group relative to the controls. Poole and colleagues [5] have argued that the reduction of depressive symptoms is a time-consuming process, since dysfunctional attitudes and negative cognitions that cause depressive symptoms tend to be more resistant to change. It is therefore possible that a reduction in depression symptoms may be possible through a longer intervention period. However, the significant associations between resilience resources and depression outcomes [81] suggest that is important to continue to focus on enhancing and reinforcing internal and external resources.

The improvements experienced by the children in the intervention group were paralleled with a reduction of trauma symptoms in parents who were involved in the activities. This may be explained by the caregiver’s adoption of self-regulatory strategies tested and acquired during the parent’s support group. Furthermore, the decrease in parents’ trauma-related symptoms is in line with other studies involving parents in resilience-focused programs for children exposed to maltreatment [40, 63]. Congruent with expectations, results showed a reduction in parental distress and a substantial increase in positive parent–child interaction, confirming our second hypothesis. The link between parental stress and negative parent–child interactions is well established in the literature [41, 82]. Parenting interventions are in fact based on the premise that children's health and development is shaped by parenting and by parent–child interaction [38]. It is therefore necessary to work on parent stress to stimulate a positive parent–child interaction [79]. In line with this research, the result obtained is further confirmation that improving family cohesion [58, 66] and communication [65] through proposing joint parent–child activities in the ToR program can reduce parental distress and increases positive parent–child interactions.

Regarding the last hypothesis that mother involvement in the ToR program would be more effective, the results show that effect sizes were greater for children whose mothers involved in the activities, for most of the trauma-related outcomes considered. In particular, children whose mothers attended the activities show a significative greater decrease in general trauma as well as in anxiety and dissociative symptoms, with a moderate effect size (*η*^2^_*p*_ = 0.04), compared to children whose mothers didn’t attend activities. These results are in line with previous studies on parent-involved programs for maltreated children, showing promising effects on reducing parental distress, increasing parental behavioral and emotional responsiveness, thus increasing child's positive affect and improved child development[42, 80].

Several studies report varying program impacts, reflecting implementation factors such as intervention goals [83], features of programmatic content and delivery [84]. This highlights the importance of detecting common practice aspects of interventions that may be ‘active ingredients’ for change in target beneficiaries [85] thus the importance of reporting differential effects for subgroups of participants [86]. Results from our study highlights the importance of recognizing the key role of family in programs for children who have experienced maltreatment and the importance of supporting parents to produce successful outcomes in children [87]. Indeed, the long-term effect of the program depends on how promoting or constraining the environments to which children return after the program [88].

However, parents’ involvement in the ToR was not mandatory, and only a proportion of caregivers decided to engage in the program. Therefore, greater efforts should be made to encourage and facilitate caregivers’ participation. In the project that is now implemented in the target centers (i.e., Teneramente per un Infanzia Felice), preliminary work has been conducted by providers in order to encourage the involvement of mothers in the center activities, and to create within the center a specific area for the caregivers to foster a mother-friendly space.

## Limitations

We must consider several limitations of the study. First, we did not have follow-up data to demonstrate the maintenance of treatment effects over time, but we relied on pre- and post-treatment comparisons as indicators of treatment effectiveness. Second, the study didn’t take the form of a randomized controlled trial, which may have led to some bias in group characteristics. Third, the absence of mothers’ control group prevented a comparison of the improvement in mother–child interactions and the decrease in mother trauma to the ToR efficacy. Further research is required to ascertain the actual efficacy of the ToR program through evidence-based studies with randomized controlled trials and to employ a larger, more representative subject sample in order to guarantee generalizability. Furthermore, a longer follow-up term, of six months, would be also necessary to assess the impact of ToR in the medium-long period. Moreover, the monitoring of comparison groups for caregivers would help to provide a more accurate view of the impact of ToR on the involved parents’ mental health and wellbeing. The inclusion of fathers and other caregivers might also help to understand the efficacy of ToR with different caregivers. This might be particularly relevant in cases of children who are separated from their parents.

Finally, the low number of mothers who completed questionnaires relative to the number of children involved in the program is a limitation of the study, and emphasizes, for future data collection, the importance of encouraging and facilitating mothers in completing questionnaires during their visit.”

## Conclusion

Resilience is a process that develops through interactions with the multiple systems that act to modify the effects of adverse life events [89, 90]. In children who have experienced maltreatment, their risk affects multiple levels of their environment; therefore efforts to enhance resilience must take place at multiple levels. Research effort has been targeted towards understanding the underlying mechanisms by which personal, family, social and environmental factors contribute to positive outcomes [91]. A growing body of research on resilience among child welfare populations suggests the need to conceptualize resilience as the result of processes associated with the actions of service providers [33, 45]. Study findings show that service providers’ implementation of the Tutor of Resilience program may lead to a significant improvement of children mental health, and more specifically reduce anxiety, dissociation, anger and PTSD. In particular, involving caregivers in the program turned out to be particularly effective for reducing parents’ trauma-related outcomes, improving parent–child interaction and for achieving greater outcomes for children. Further research is also needed to explore the relationship between the child and its environments and to explain the nature of their direct and indirect effects on one another. This knowledge can greatly improve the development of programs.

## Implications

A proper understanding of risk and resilience is essential to the design and implementation of policies and programs that attempt to acknowledge the effects that maltreatment can have on children. In particular, the role of a supportive family in the development of resilience in children is of the utmost importance. It is therefore fundamental that interventions should be designed to nurture resilience-promoting competencies and resources at the family level, thereby enhancing family functioning and positive interactions within the family. At the same time, building resilience requires a focus on both the provision of opportunities to access protective factors in the social environment and change social environments to further promote wellbeing [92]. In particular, social providers, if properly trained, can serve as effective resilience-building actors. The ToR is an effective resilience-building program that can guide service providers in the creation of contextually sensitive interventions in ways that both mitigate risk and enable access to resilience-promoting resources for children, youth and families experiencing various forms of adversity, thereby enhancing their psychosocial well-being.

## Summary

Resilience is conceptualized as a dynamic process that involves drawing on both internal and external resources to achieve positive outcomes despite adversity. Such processes are facilitated by protective relationships and physical ecologies that make resources available and accessible in ways that individuals experience as meaningful. Studies of resilience have focused on understanding its underlying mechanisms in children who experience maltreatment and to explore the impact of resilience-building interventions on improving wellbeing. A growing number of studies have also indicated the benefit of including parents in resilience-informed child support programs to improve individual outcomes in both parents and children as well as increasing positive child-parent interactions. This study aims to investigate the impact of the Tutor of Resilience (TOR) program—a resilience-building intervention for both children and caregivers—with a group of Italian children who have experienced maltreatment, as well as the differential program effects of mothers’ involvement. Participants were 186 children (mean age = 11.95; SD = 2.50) who had experienced different types of maltreatment and who had been referred to one of three day-care centers. Day-care center providers received training in the TOR program. Parent- and child-reports of trauma symptoms and parent reports of the parent–child relationship were assessed at baseline and 10 months later at the end of the program. A control group was involved which consisted of 88 children (mean age = 10.76; SD = 2.57) reporting similar experiences of maltreatment. Repeated-measures ANOVA was used to contrast potential improvements over time in trauma symptomatology and in mother–child interaction between the intervention and control groups, as well as to compare trauma-related outcomes in children whose parents were involved in the intervention with those who were not. Children involved in the ToR program reported a significant reduction in trauma-related symptoms compared to the control group, in terms of anxiety, anger, post-traumatic stress, and dissociation, while mothers reported a significant improvement in their own general trauma symptoms, their distress, and interactions with their child. Furthermore, children whose mothers were involved in the ToR activities also demonstrated significantly greater improvements in child-reported symptoms of anxiety and dissociation, compared with children whose mothers were not involved. Findings support the effectiveness of the ToR program as delivered by day-care providers for children who experienced maltreatment, especially when the mother of the child is involved in intervention activities.

## Footnotes

1 The ToR program was conducted in collaboration with the CESVI foundation.
